# Smaller islands, bigger appetites: evolutionary strategies of insular endemic skinks

**DOI:** 10.1098/rsos.240870

**Published:** 2024-10-02

**Authors:** Catarina J. Pinho, Herculano A. Dinis, Brent C. Emerson, Raquel Vasconcelos

**Affiliations:** ^1^ CIBIO-InBIO, Centro de Investigação em Biodiversidade e Recursos Genéticos, Laboratório Associado da Universidade do Porto, Campus Agrário de Vairão, Vairão, Portugal; ^2^ Departamento de Biologia, Faculdade de Ciências da Universidade do Porto, Porto, Portugal; ^3^ BIOPOLIS Program in Genomics, Biodiversity and Land Planning, CIBIO, Campus de Vairão, Vairão, Portugal; ^4^ Associação Projecto Vitó, São Filipe, Ilha do Fogo, Sao Filipe, Cabo Verde; ^5^ IPNA-CSIC, Island Ecology and Evolution Research Group, Institute of Natural Products and Agrobiology, Tenerife, Spain

**Keywords:** Cabo Verde, competition, resource partitioning, morphology, DNA metabarcoding, reptiles

## Abstract

Competitive dietary and morphological divergence among co-occurring species are fundamental aspects of ecological communities, particularly on islands. Cabo Verde (~570 km west of continental Africa) hosts several endemic reptiles descended from common ancestors, with sympatric species exhibiting wide morphological variation and competing for limited resources. To explore the mechanisms of resource partitioning between coexisting species, DNA metabarcoding was used to compare the diets of large and small skinks, *Chioninia vaillantii* and *Chioninia delalandii*, in sympatric and allopatric contexts on Fogo Island and in a more competitive context on the small and resource-poor Cima Islet. The morphological variation of all populations was also examined to test the character displacement hypothesis and to compare the effect of different competitive scenarios. Results showed significant differences in diet and linear measurements between species and populations. The two sympatric populations of *C. delalandii* on Fogo and Cima showed similar changes in head morphology compared to the allopatric population, supporting character displacement. The effect of higher competitive pressure on Cima was evidenced by the increased morphological and dietary variation observed. This study demonstrates how sister species develop dietary adaptations/morphologies to maintain stable coexistence, especially in highly competitive scenarios, providing useful insights for effective conservation strategies.

## 1. Introduction

Competition plays a fundamental role in shaping ecological communities, primarily by driving dietary segregation between coexisting species [1,2]. This is particularly expected among closely related species, as they tend to have similar morphologies, feeding habits, and distribution patterns [3]. Thus, competition between syntopic species should be marked in resource-poor habitats [4,5]. Traits involved in food exploitation are expected to come under greater evolutionary pressure [5]. Therefore, understanding dietary niche partitioning and associated morphological divergence between evolutionarily and ecologically related co-occurring species is important for developing a general knowledge of community assembly and coevolution [6,7]. Ultimately, this helps to predict how ecosystems might respond to increased pressures, such as prey extinction or the introduction of alien species.

Competition-driven phenotypic specialization has been observed in several vertebrates, including birds [8], fishes [9] and reptiles [10]. In the latter, variations in body, head and limb sizes have been shown to provide functional advantages to competing species (e.g. [11]). These contribute to microhabitat segregation and are highly relevant in social interactions, such as mating [12–14]. Notably, head shapes play a direct role in dietary specialization [6,10]. Larger heads have been shown to result in stronger jaws, and increased bite forces [6,10], this generally allowing the consumption of larger/harder prey and more plant matter [10,15]. Moreover, variations in limbs allow species to occupy different microhabitats and foraging niches [16,17]. Differences in locomotor capability may lead to dietary segregation due to disparities in prey capture ability [18].

**Figure 1.**
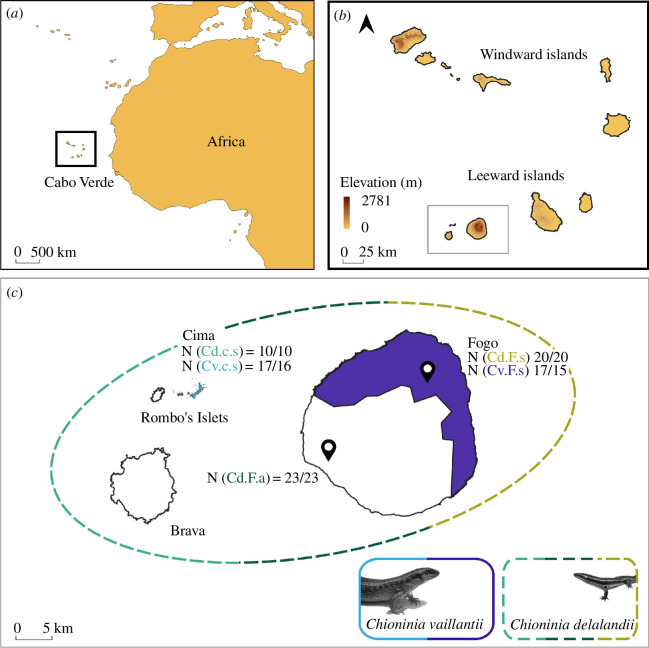
Study area and taxa. (*a*) Geographic location of Cabo Verde (Geographic Coordinate System, Datum WGS84). (*b*) Island elevation and groups: Windward (Santo Antão, São Vicente, Santa Luzia, São Nicolau, Sal and Boavista) and Leeward (Maio, Santiago, Fogo, Brava and Rombo’s). (*c*) Geographical distributions of the *Chioninia* populations (Cd, *C. delalandii* in green tones; Cv, *C. vaillantii* in blue tones). Skinks are represented showing their size discrepancies. Dashed lines represent the species with the largest distribution and filled areas correspond to sympatric areas. The number of faecal samples (*n*) extracted, and successfully sequenced, respectively, are also shown for each of the five populations (F, Fogo Island; c, Cima Islet; s, sympatric; a, allopatric).

Islands offer unique opportunities to study the impact of competition on ecological and evolutionary processes, such as morphological and dietary specialization [11,19]. Small islands are particularly interesting, harbouring endemics with unique morphological and behavioural adaptations [20,21]. Comprehensive studies on niche partitioning strategies among syntopic sister species are greatly facilitated within systems where it is possible to characterize and compare dietary habits and morphological variation between sympatric and allopatric populations. This framework, which exists in the Cabo Verde Archipelago, allows understanding the extent to which the presence/absence of direct competition influences species coexistence. These ten oceanic islands and several islets lie within Macaronesia (figure 1*a,b*) and within the Mediterranean Basin biodiversity hotspot [22]. They host a remarkable diversity of terrestrial reptiles, including 32 endemic taxa [23,24], such as the genus *Chioninia* Gray, 1845 [25–27]. Before phylogenetic studies, this genus was synonymous to *Mabuya* Fitzinger, 1826 and the monospecific *Macroscincus* Bocage, 1873, which included the giant Cocteau’s lizard [25,28,29]. *Chioninia* skinks originated from a single colonization event from continental Africa, around 26 million years ago (Mya) [30], originating six extant species and seven subspecies, and one extinct species, *Chioninia coctei* Duméril & Bibron, 1839 [27].

An increased rate in the evolution of body size has been demonstrated for the genus [30]. Based on phylogenetic reconstruction, most *Chioninia* species have retained the medium body size of the ancestor [31], such as *Chioninia delalandii* (Duméril & Bibron, 1839), but others have evolved larger sizes, such as *Chioninia vaillantii* (Boulenger, 1887) [27]. The latter is represented by two subspecies, *C. vaillantii vaillantii* on Santiago Island and *C. vaillantii xanthotis* on Fogo Island and Rombo’s Islets [27]. These two sister species are syntopic throughout their ranges with the smaller *C. delalandii*, also found in allopatry and widespread on the Leeward islands of Santiago, Fogo, Brava, Maio (introduced), and Rombo’s [23,27]. These species are estimated to have diverged around 6.9−5.9 Mya [27] and are both diurnal (figure 1*c*) [23], potentially competing for trophic resources.

To explore resource partitioning between these coexisting species, DNA metabarcoding was used to characterize and compare the diet composition of both *Chioninia* species in three contexts (figure 1*c*): (i) on Fogo, where both species are sympatric in humid uplands with abundant vegetation, (ii) an allopatric population of *C. delalandii* inhabits lowland areas, and (iii) on Cima Islet, where both species are sympatric and competitive pressures are expected to be higher, with mice reaching high densities and having a major impact on limited *Chioninia* resources [32]. Additionally, linear measures were compared among populations to explore how ecological and competitive pressures associated with species coexistence have shaped body morphologies. Populations on Cima are expected to show higher divergence in diet and morphology than the more relaxed ones on Fogo. According to optimal foraging theory [33], with lower availability of preferred resources an expansion of the dietary niches of these populations is expected. This would translate into both species exhibiting a more generalist and diverse diet on Cima when compared to Fogo. Also, a less pronounced diet overlap is expected, evidence of resource partitioning, and greater morphological divergence on Cima when compared to sympatric and allopatric species on Fogo. This should be particularly evident in head morphology, strongly associated with adaptations to different resources. This setup provides a suitable framework to investigate evolutionary responses to competitive pressures, such as testing the hypothesis of character displacement—the evolutionary process whereby two resource competitors diverge in phenotype and resource utilization, facilitating coexistence [34]—leading to disruptive selection and maintenance of the competing species. Moreover, studying these processes can help to develop sustainable conservation strategies, for instance by identifying the essential food items for the survival of both species.

## 2. Methods

### 2.1. Study area

Fogo Island and Rombo’s Islets are located in the Leeward group of Cabo Verde (figure 1*b*). Fogo has an area of 476 km^2^, reaching the highest altitude of the country at 2829 m [35]. The landscape is characterized by an active volcano, last erupted in 2014 [36], and large agricultural and pasture areas within its wide caldera. Its climate varies from dry—in the lower areas—to sub-humid—in higher altitude areas [37]. In 2020, it was declared a biosphere reserve by UNESCO due to its unique ecosystem and biodiversity [38].

The uninhabited Rombo’s are composed of two main islets, Grande and Cima, surrounded by the smaller islets of Luiz Carneiro, Rei and Sapado (<0.25 km^2^). These are classified as Integral Nature Reserve [39] and constitute Important Bird Areas [40]. Cima, approximately 15 km west of Fogo, is a dry flat islet of about 1.5 km^2^ and a small hill of approximately 77 m. This islet is a key biodiversity hotspot for seabird species [32,41]. However, its biodiversity is severely affected by *Mus musculus* Linnaeus, 1758 increasing populations [32].

### 2.2. Study species

Delalande’s skink *C. delalandii* (Cd hereon) is medium-sized (adults between 52 and 92 mm snout–vent length, SVL), and easily recognized by the presence of a black dot on the axilla and yellow eyelids [27]. It is often found in stonewalls and beneath rocks within agricultural and livestock areas [42]. On Fogo, it is also found in urban areas, including at higher altitudes (figure 1*c*) [23]. Despite its abundance and conspicuousness [43], little is known about its ecology, except for anecdotal reports of omnivory [44]. It is classified as Least Concern according to the International Union for the Conservation of Nature (IUCN) Red List of Threatened Species [43], and as Low Risk on the National Red List at a national and main islands level, but Data Deficient on Rombo’s [45].

The Vaillant’s skink of Fogo *C. vaillantii xanthotis* (Cv hereon) is large-sized (adults between 87.5 and 105 mm SVL), and distributed on Fogo and Rombo’s, more precisely on Cima [23]. It can be identified by its three well-contrasted stripes along the body and the bright yellow scales around the ear opening [27]. On Fogo, it is restricted to the northwestern side (figure 1*c*) and less abundant than Cd. It is typically found in stonewalls at high altitudes in vegetated humid habitats [23]. It is viviparous and its diet is thought to be composed mostly of insects and plants [44]. It is classified as Endangered according to the IUCN [46], as Undetermined on a national level and on Fogo and as Data Deficient on Rombo’s in the National Red List [45].

### 2.3. Sampling

**Figure 2.**
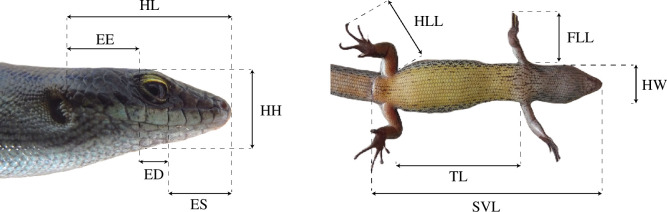
Linear measures taken for both *Chioninia* species. The measurements of the head included head length (HL), width (HW), and height (HH), eye–snout distance (ES), eye–ear distance (EE), and eye diameter (ED). Body measurements included snout–vent length (SVL), trunk length (TL), and front (FLL) and hind limb length (HLL). The photos given as examples correspond to specimens of *Chioninia delalandii* (photographs by authors).

Sampling took place on Fogo and Cima in May 2019, with additional samples of Cd collected on Fogo in September 2021 in the allopatric area. On Fogo, 20 samples of Cd were collected in sympatric areas and 23 in allopatric areas, and 17 of Cv in sympatry. On Cima, a total of 10 and 17 faecal samples of Cd and Cv were collected, respectively. Specimens were captured using either hoop fishing or bucket traps with bait (banana and mango). Faecal samples were collected by abdominal massage and immediately preserved in 96% ethanol tubes. These were stored at −20°C until further processing. Individuals were sexed, photographed and geolocated using a GPS device. Before release, morphological linear measures of both species were taken to the nearest 0.1 mm using digital callipers (figure 2): SVL, trunk length (TL), head length (HL), width (HW), and height (HH), eye–snout (ES) and eye–ear (EE) distance, eye diameter (ED), and front (FLL) and hind limb length (HLL). Animals were placed in separate opaque tissue bags while others were processed to reduce stress.

In addition, pitfall traps with water were placed close to the areas where skinks were captured to collect invertebrates to build a DNA reference database of available food items. Captured specimens were preserved in falcon tubes with 96% ethanol until examination under a magnifying lens with an assembled camera. Individuals were sorted into different high-level taxonomic groups based on morphological identification and photographed for higher-resolution identification by experts.

### 2.4. Diet analysis

#### 2.4.1. Library preparation

DNA from faecal samples was extracted using a Stool DNA Isolation Kit, following the manufacturer’s instructions. Each batch of 23 samples included an extraction negative control. Extracted DNA was amplified using previously validated markers [47] for three diet groups: invertebrates with the IN16STK-1F-mod/IN16STK-1R-mod primers, targeting the mitochondrial 16S gene [47,48]; plants with g/h primers targeting the short P6-loop of chloroplast *trnL* (UAA) gene [49]; and vertebrates with the 12sv5F/12Ssv5R primer pair, targeting V5-loop within the mitochondrial 12S gene [50]. To prevent the amplification, with the 12S primers, of host DNA, a blocking primer was designed (5′-TCCTCTAGGTCGGTATGGGGCACCGCCA(C3 spacer)-3′). For this, 12S sequences of both *Chioninia* species were obtained from GenBank and aligned with sequences from birds, fish and reptiles known to occur in Cabo Verde. Its effectiveness for Cd and Cv was validated by comparing PCR amplification with and without the blocking primer, using previously extracted DNA from tail tissues. PCR products (see details in electronic supplementary material) were cleaned using Agencourt AMPure XP beads, quantified using Nanodrop, diluted to 15 nM, and pooled for each marker. The three libraries were then quantified using qPCR and pooled to obtain a final library at 4 nM, which was sequenced using the MiSeq Reagent Kit V2 for an expected average of 20 000 paired-end reads per PCR product.

Invertebrate DNA for the reference library was extracted from legs or whole individuals (when very small), using saline extraction methods following [51] and amplified using two primer sets: the IN16STK-mod primers, which follow the previously described methods and allow matching to dietary items, and the LCO1490/HC02198 primers, which target the animal barcode region of cytochrome oxidase I (COI). PCR conditions used for the latter are described in previous studies [52]. Barcode sequencing was undertaken to assist with taxonomic assignments.

#### 2.4.2. Bioinformatics filtering

Sequences were bioinformatically processed using the OBITtools software [53], which in summary performs the alignment of the sequences obtained and the filtering of PCR/sequencing errors to obtain molecular operational taxonomic units (MOTUs; see details in electronic supplementary material). The LULU R package was used to combine MOTUs with >84% similarity [54], removing remaining errors. Based on PCR blank counts, PCR products with less than 2000 reads were removed and within the remaining samples, MOTUs representing less than 1% of the total reads of that PCR product were also excluded. The remaining MOTUs were then compared against the NCBI Nucleotide Database and our reference collection, using the BLAST+ software, and classified to the lowest possible taxonomic rank. If a sequence matched more than one species with equal similarity values all belonging to the same genus, a genus-level assignment was considered. When a sequence matched more than one species or genus with similar probabilities, only species or genera known to occur in the archipelago were considered, otherwise a higher ranking would be attributed (e.g. family). If different MOTUs corresponded to the same taxon, a number would be attributed to each one of them (e.g. Araneae_1; Araneae_2). MOTUs detected in extraction blanks, considered as contaminations or of sampling baits (banana and mango) were removed from the corresponding batch of samples.

#### 2.4.3. Data analysis

The frequencies of occurrence (FO) of each of the three diet item groups (plant, invertebrate, vertebrate) were estimated for the sympatric populations from Fogo (Cd.F.s and Cv.F.s from here on) and Cima Islet (Cd.c.s and Cv.c.s from here on), and the allopatric population of Cd from Fogo (Cd.F.a from here on). To test for differences in proportions of each diet item group between populations, FOs were compared using two-proportion z-tests with the function *prop.test* in R software version 4.3.2 [55].

Family level identification was used for the analysis due to the inability to achieve species-level taxonomic resolution for several MOTUs. To visualize the dietary networks at the family level for the five populations, bipartite networks were generated using the function *plotWeb* from bipartite package v2.19 [56]. Adjacency plots were calculated at the order level using the function *metaComputeModules* and visualized using *plotModuleWeb*. Rarefaction curves were used to compare resource richness at the family level among the five populations. Richness values were estimated for the double of species with the lower sample size and differences were considered significant if the 95% confidence intervals did not overlap [57]. The functions *iNEXT* and *ggiNEXT* from the INEXT package [58] were used to perform and visualize this analysis, respectively.

Dietary niche overlap within the two sympatric populations (Cd.F.s versus Cv.F.s and Cd.c.s versus Cv.c.s) was calculated using Pianka’s index at the family level [59]. This index ranges from 0 to 1, where 0 indicates no common resources and 1 indicates complete resource overlap. To test whether the dietary niche overlap differed significantly from what would be expected by chance, null models were generated based on the RA3 randomization algorithm [60], generating 10 000 null matrices that were compared to observed data, using the EcoSimR package [61]. Non-metric multidimensional scaling (NMDS) was used to evaluate prey-use differences among populations. Samples were ordinated in a two-dimensional space according to diet dissimilarity, using the function *metaMDS* from the vegan package [62] and based on a Jaccard distance matrix.

Finally, generalized linear models (GLMs) were performed to test for significant differences in diet composition at the MOTU, family and order levels in the following contexts: within sympatric species (Cd.F.s versus Cv.F.s and Cd.c.s versus Cv.c.s); between species from different islands (Cd.F.s versus Cd.c.s and Cv.F.s versus Cv.c.s); and between Cd allopatric populations (Cd.F.s versus Cd.F.a). GLMs for multivariate presence/absence data were fitted by implementing the *manyglm* function of the *mvabund* package [63]. The complementary log–log distribution provided the best fit and was used for subsequent tests. The significance of the GLMs was tested using the *anova.manyglm* function that was then implemented with the argument *p.uni= 'adjusted'* to perform univariate tests for identifying prey items responsible for differences in diet composition between species and among populations.

### 2.5. Morphological analysis

Morphological measures (electronic supplementary material, table S1) were log-transformed to ensure the normality of the data distribution. A principal component analysis (PCA) of all measurements was performed, using the function *prcomp* from the package *stats* in R software [55], taking into account all populations and both sexes. Analyses of variance (ANOVAs) were performed, using the *adonis2* function of the *vegan* package [62], to test for the possibility of sexual dimorphism within the study species, as well as to examine potential differences in total body size among the five populations, represented by SVL.

Before further significance testing, size effects were removed for all variables, given the larger size of Cv when compared with Cv, using the *allom* function from the package GroupStruct [64]. All individuals were scaled to the same size and their shape was adjusted to the new size according to the allometry principle [65,66]. A new PCA was performed on the size-adjusted values and a new plot was generated showing the contribution of each variable to the observed variances. ANOVAs were performed using each of the adjusted linear parameters as response variables, and the populations as independent variables. *Post hoc* Tukey’s honest significant difference (HSD) tests [67] were performed on the ANOVAs, using the function *TukeyHSD* from the package *stats* [55], showing how populations differed from each other.

## 3. Results

### 3.1. Diet composition

The final dataset comprised 84 samples (Cd.F.s = 20, Cv.F.s = 15, Cd.F.a = 23, Cd.c.s = 10, Cv.c.s = 16) with an average of 16 347 reads per sample. A total of 144 MOTUs of nine taxonomic classes, 40 orders and 88 families were identified (electronic supplementary material, table S2). On Fogo, Fabaceae was the most frequent family identified in the diets together with Malvaceae for Cd.F.s, Convolvulaceae for Cv.F.s and Blattidae for Cd.F.a (electronic supplementary material, figure S1). On Cima, the most frequent families in the diets were Formicidae and Tenebrionidae for Cd.c.s and Zygophyllaceae and Caryophyllaceae for Cv.c.s (electronic supplementary material, figure S1).

Plant frequency was similar across all populations, except for Fogo allopatric populations, which presented significantly lower values (FO_Cd.F.s_ = 100.00% versus FO_Cd.F.a_ = 52.00%, χ^2^ = 10.46, *p* = 0.00). Invertebrate frequencies showed significant differences between islands and populations, with Fogo populations presenting lower values than on Cima (FO_Cd.F.s_ = 15.00% versus FO_Cd.c.s_ = 100%, χ^2^_Cd.F.s vs. Cd.c.s_ = 16.30, *p* = 5.38 × 10^−5^; FO_Cv.F.s_ = 20.00% versus FO_Cv.c.s_ = 69.00%, χ^2^_Cv.F.s vs. Cv.c.s_ = 15.50, *p* = 0.02), and sympatric population presenting lower values than allopatric ones on Fogo (FO_Cd.F.s_ = 15.00% versus FO_Cd.F.a_ = 100.00%, χ^2^ = 28.87, *p* = 7.72 × 10^−8^). Vertebrates were only present in the diet of the sympatric Cima populations (FO_Cd.c.s_ = 30.00%; FO_Cv.c.s_ = 25.00%) and the allopatric population of Fogo (FO_Cd.F.a_ = 9.00%).

**Figure 3.**
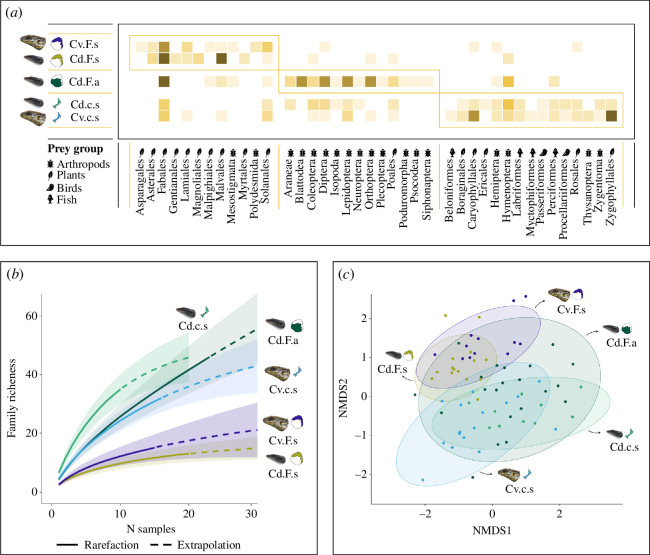
Diet composition results for the five *Chioninia* populations (Cd, *C. delalandii*; Cv, *C. vaillantii*; F, Fogo Island; c, Cima Islet; s, sympatric; a, allopatric). (*a*) Adjacency matrix summarizing the population subgroups according to their identified diet item orders. The species are represented proportionally according to their sizes along the vertical axis, and plant, arthropod and vertebrate orders are shown along the horizontal axis. Subgroups are delineated by yellow boxes. The darker the squares the more frequent the association between a diet item and its predator. (*b*) Accumulation curves depicting observed (full line) and extrapolated (dashed line) family richness with their 95% confidence intervals. (*c*) Non-metric multidimensional scaling ordination (NMDS) of the diet composition at the family level. Points closer together correspond to samples with a more similar dietary composition. Ellipses represent the standard error of the dietary composition centroids at 95% confidence intervals.

The adjacency matrix defined three modules at the order level, grouped by location (figure 3*a*). The sympatric species on Fogo (Cd.F.s and Cv.F.s) showed significantly lower richness values compared to the other three populations (figure 3*b*). Dietary niche overlap (O) at the family level was similar for both sympatric comparisons and not significantly different than expected by chance (O_Cd.F.s vs. Cv.F.s_ = 0.580; O_Cd.c.s vs. Cv.c.s_ = 0.530). The NMDS showed a generally moderated diet overlap within sympatric populations (figure 3*c*).

Multivariate analyses revealed significant differences in the diet composition of all populations at the MOTU, family, and order levels (*p* = 0.00 for all). Pairwise comparisons were also significant, except for the comparison Cd.c.s versus Cv.c.s at the order level (electronic supplementary material, table S3). Univariate tests showed differences in diet composition due to seven MOTUs: *Paronychia illecebroides* Webb (*p* = 0.001), exclusively consumed by the sympatric populations of Cima (Cd.c.s and Cv.c.s; electronic supplementary material, table S2); *Sida acuta* Burm.f. (*p* = 0.002), *Cajanus cajan* (L.) Millsp. (*p* = 0.004), *Phaseolus*_1 (*p* = 0.006) and *Desmodium scorpiurus* (Sw.) Poir. (*p* = 0.041), exclusively consumed by the sympatric populations of Fogo (Cd.F.s and Cv.F.s; electronic supplementary material, table S2); and *Periplaneta americana* (Linnaeus, 1758) (*p* = 0.004), exclusively consumed by Cd.F.a (electronic supplementary material, table S2). The families that contributed significantly to these differences were Caryophyllaceae (*p* = 0.001), Malvaceae (*p* = 0.001), Blattidae (*p* = 0.002), Muscidae (*p* = 0.004) and Asteraceae (*p* = 0.046; electronic supplementary material, table S2 and figure S1). The orders Blattodea (*p* = 0.001), Caryophyllales (*p* = 0.001), Malvales (*p* = 0.001), Hymenoptera (*p* = 0.003), Diptera (*p* = 0.004), Lepidoptera (*p* = 0.008), Araneae (*p* = 0.021), Orthoptera (*p* = 0.029), Coleoptera (*p* = 0.039), Asterales (*p* = 0.042) and Fabales (*p* = 0.044) contributed significantly to these differences. The results of the univariate tests for specific population pairwise comparisons are presented in electronic supplementary material, table S4.

### 3.2. Morphological analysis

There were no significant differences between sexes (*p* = 0.14); therefore, all subsequent analyses were performed combining them. PCA of unadjusted measures showed a clear division between both species due to the expected significant differences in SVL (*p* = 0.00; electronic supplementary material, figure S2A). There were significant differences in SVL between the two sympatric populations of Cd from the different islands (Cd.F.s versus Cd.c.s: *p* = 0.023; electronic supplementary material, figure S2A).

**Figure 4.**
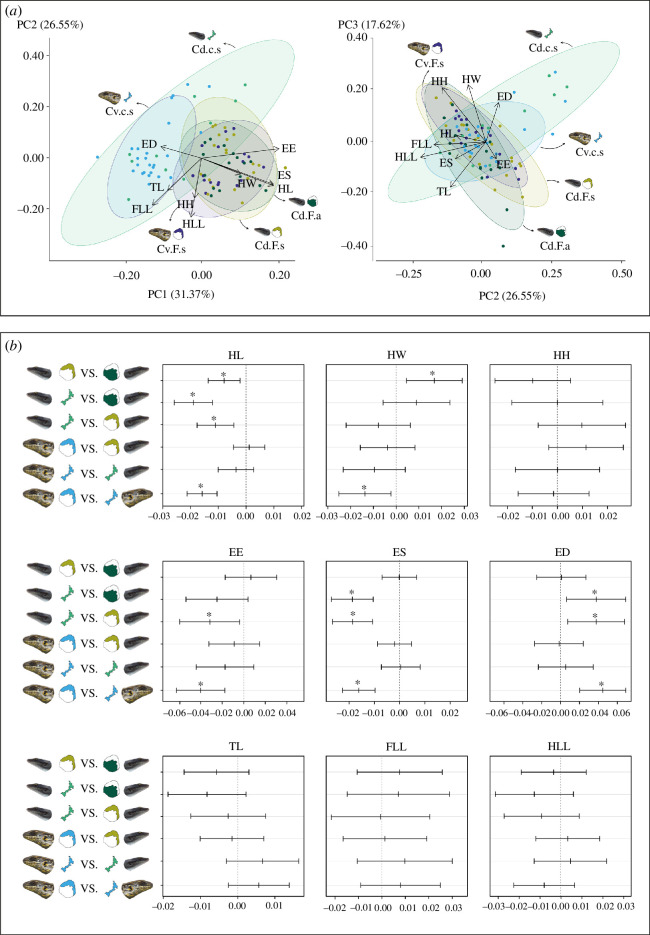
Morphological analysis results considering the snout–vent length (SVL) adjusted values for the five *Chioninia* populations (Cd, *C. delalandii*; Cv, *C. vaillantii*; F, Fogo Island; c, Cima Islet; s, sympatric; a, allopatric). (*a*) Principal component analysis (PCA) where individuals are represented by a circle and ellipses represent the 95% confidence intervals. The contribution of the variables for the PCs is also represented. (*b*) Results from Tukey’s honest significant difference (HSD) tests (HL, head length; HW, head width; HH, head height; ES, eye–snout distance; EE, eye–ear distance; ED, eye diameter; TL, trunk length; FLL, front limb length; HLL, hind limb length) among populations. The horizontal bars represent the 95% confidence intervals for each comparison and the asterisk (*) indicates the significant differences in mean levels among populations.

After size adjustment, the PCA showed a clear separation along PC1 between the populations of the two islands (figure 4*a*). This axis explained about 31.00% of the variance in the dataset and was associated with variation in EE, ES and HL (figure 4*a*). PC2 explained about 27.00% of the variation and was mostly associated with variation in the limbs (HLL and FLL) and HH (figure 4*a*). Finally, PC3 explained about 18.00% of the variation and was associated with HW, HH, TL and ED (figure 4*a*).

The Tukey HSD tests showed several significant differences between the pairs of populations across the size-adjusted linear measures (figure 4*b*). Regarding head variables, HL showed significant differences in the pairs Cd.F.s versus Cd.F.a (*p* = 0.002), Cd.c.s versus Cd.F.a (*p* = 3.85 × 10^−10^), Cd.c.s versus Cd.F.s (*p* = 0.000) and Cv.c.s versus Cv.F.s (*p* = 1.330 × 10^−10^), with both species showing lower values for Cima populations (figure 4*b* and electronic supplementary material, figure S2B). HW showed significant differences in the pairs Cd.F.s versus Cd.F.a (*p* = 0.002) and Cv.c.s versus Cv.F.s (*p* = 0.010), with Cd.F.a and Cv.c.s showing lower values (figure 4*b* and electronic supplementary material, figure S2B). Considering ocular and ear variables, EE showed significant differences between the pairs Cd.c.s versus Cd.F.s (*p* = 0.02) and Cv.c.s versus Cv.F.s (*p* = 3.43 × 10^−5^) and ES and ED showed significant differences between Cd.c.s versus Cd.F.a (*p*_ES_ = 1.21 × 10^−7^; *p*_ED_ = 0.01), Cd.c.s versus Cd.F.s (*p*_ES_ = 4.59 × 10^−8^; *p*_ED_ = 0.008) and Cv.c.s versus Cv.F.s (*p*_ES_ = 6.29 × 10^−9^; *p*_ED_ = 2.85 × 10^−5^; figure 4*b*). These results are explained by the lower values of both Cima populations for these variables (electronic supplementary material, figure S2B).

## 4. Discussion

The observed differences in dietary composition and morphological traits between sympatric and allopatric populations of *C. delalandii* provide strong evidence for character displacement as an evolutionary strategy that has facilitated stable coexistence with *C. vaillantii.* Furthermore, increased competitive pressure on Cima Islet is associated not only with a significantly different diet composition but also with morphological variation between the two species, compared to their counterparts on Fogo. As predicted, with increased trophic competition, the traits that showed significant differences were those involved in the exploitation of food resources, such as head size. This supports the existence of greater evolutionary pressure on these traits underpinning stable coexistence.

### 4.1. Diet composition comparisons

As initially predicted, both species from Cima Islet present more generalist diets, consuming all food groups, while the diet of Fogo sympatric populations is mainly composed of plants. A striking difference between the islands is the presence of vertebrates in the diet of Cima population, the first time this is reported for these species. This dietary shift may indicate an adaptation to the limited resources on the islet, leading to the predation of more nutritionally valuable items, as previously seen in other reptile species of Cabo Verde, such as geckos [47], and from other Macaronesian islands, such as lacertids in the Azores [68] and the Selvagens gecko [69]. The presence of fish items in the diet is most likely a result of the ingestion of seabird regurgitations and/or faeces, as evidenced by the presence of *Pelagodroma marina* (Latham, 1790) and *Bulweria bulwerii* (Jardine & Selby, 1828). Additionally, fish may have originated from leftovers from fishing activity, reflecting opportunistic feeding on vertebrates. The allopatric *C. delalandii* population on Fogo also presented vertebrates in its diet, particularly fish, albeit at low frequencies. This is certainly related to anthropogenic waste, as exemplified by the presence of *Selar crumenophthalmus* (Bloch, 1793), a widely consumed fish in Cabo Verdean cuisine. In general, species on Cima consumed more native plants (e.g. *Zygophyllum simplex* L. in *C. vaillantii*’s diet) than Fogo populations, which consumed more crops (e.g. *Sida acuta* Burm. f. exclusively present in the diet of *C. delalandii* sympatric population). The significant differences between the two *C. delalandii* populations on Fogo are the result of more plants and less invertebrates present in the diet of the sympatric comparing to the allopatric population. This is consistent with a niche shift in the presence/absence of a direct competitor, similar to what has been observed with another endemic Cabo Verdean reptile *T. raziana* [70].

Both species on Cima showed significantly greater dietary richness, along with the *C. delalandii* allopatric population, when compared to the sympatric species on Fogo. These findings align with optimal foraging theory, which postulates a decrease in the availability of preferred resources associated with an expansion of the dietary niche [33]. On Cima, given the low resource availability, the preferred food items of both species are very restricted. This, coupled with interspecific competition (skinks and mice), may have compelled individuals to broaden their diets, incorporating previously overlooked items [71]. This is consistent with an adaptive response to cope with competitive pressures in the face of scarcity [72]. In contrast, Fogo sympatric populations are expected to have greater access to preferred resources, facilitating a narrower diet niche. Conversely, the richer diet observed in the allopatric population of *C. delalandii* on Fogo may indicate that these individuals have the opportunity to diversify their diet in the absence of direct competition [73,74].

Contrary to the initial hypothesis, dietary niche overlap values at the family level were similar for both species in sympatry on Fogo and Cima. Both pairs showed moderate dietary overlap; however, these results were not significantly different from what would be expected by chance and should be further explored. Despite this, sympatric species on both islands share around a quarter of the MOTUs and dietary items at the family level. This, coupled with the significantly different diet compositions within sympatric species, highlights the distinct exploitation of the resources by the two species. In particular, on Fogo, small and large species exhibit distinct feeding patterns on invertebrates, with no shared MOTUs. On Cima, the observed significant differences were mainly due to the consumption of Tenebrionidae, exclusively consumed by *C. delalandii*. This suggests that a stable coexistence is already established with dietary niche segregation as a mechanism through which competition is avoided between both species in both areas [75].

### 4.2. Morphological comparisons

The first striking outcome from the morphological analysis is the divergence in overall body morphology between the two sympatric scenarios on Fogo and Cima. The significantly smaller body size of *C. delalandii* from Cima compared to the sympatric population from Fogo may indicate phenotypic plasticity in response to harsh habitat conditions [76] or may already reflect selective forces acting on this population. Given the low resource availability of Cima and direct competition with the larger skink and mice, this medium-size skink may need to adopt more active foraging and predation techniques and reduce food intake needs. Insectivorous and omnivorous species tend to be smaller than herbivorous species because they need to be more agile to hunt active prey [6]. This is consistent with the diet results, showing a more omnivorous diet on Cima and a more herbivorous diet on Fogo. Variations in body size can affect other characteristics such as reproduction and thermoregulation [77,78]. Smaller species usually exhibit shorter gestation periods and times, reducing reproductive energy costs [77]. Smaller reptiles can absorb and dissipate heat more rapidly than larger ones, enhancing their ability to regulate body temperature faster [78,79]. Both of these characteristics are very advantageous for populations facing suboptimal conditions (e.g. [80]).

Regarding size-adjusted head dimensions, both sympatric *C. delalandii* populations showed significantly shorter and wider heads in comparison with the allopatric *C. delalandii* population. Wider and shorter heads in reptiles are usually associated with more plant-eating or more diverse diets [6]. A wider head creates additional space for jaw adductors, potentially contributing to an enhanced bite force [6,81]. In contrast, longer heads provide faster yet relatively weaker bites [6]. The two sympatric *C. delalandii* populations significantly differed in HL, with the population from Cima showing shorter heads. This latter also presented significantly shorter snouts, suggesting an adaptation to further increase the mechanical advantage of the jaw without increasing muscle forces [82]. These results are consistent with the presented dietary results of *C. delalandii*, as the allopatric population is more dominated by invertebrates, presenting longer and narrow heads; a more plant-based diet of the sympatric population from Fogo, thus shorter and wider heads; and a more generalist diet of the sympatric population from Cima, reflected in even shorter heads. Similar patterns of head size variation are observed between the two *C. vaillantii* populations. The Cima population shows significantly shorter heads and snouts, while the Fogo population shows wider heads. This is likely associated with the observed broader diet of the Cima population and a more plant-focused diet on Fogo. Wider heads may allow the effective reduction of fibrous plant material before swallowing, facilitating digestion [83]. The head morphology of the Cima population likely allows a more omnivorous and diverse diet [6,15] in response to the low resource availability in the islet.

Significant differences were also observed in the ED, with both species from Cima presenting larger eyes than Fogo populations. This divergence indicates an adaptation associated with the activity period, with larger eyes associated with a more nocturnal activity [84]. Both are typically diurnal species [42,44], but on Cima they developed larger eyes, enhancing low light sensitivity, as an adaptive strategy to increase their temporal window for prey capture [85]. This allows them to hunt prey during twilight when nocturnal invertebrates are more active. Several MOTUs of nocturnal moths were exclusively found in the diet of the two species (e.g. Gelechiidae_1 and Noctuidae_1), further supporting this. Additionally, larger eyes improve focal resolution which improves the ability to detect prey [84,85]. This adaptation helps increase their overall foraging efficiency and meet nutritional requirements in this resource-constrained islet, in contrast with the Fogo populations which do not face these challenges. Larger eyes occupy more space within the head, which explains the significant reduction in the eye–ear distance in the Cima species. This translates into smaller jaw adductor muscles correlated with a less powerful bite [82]. However, these populations are compensating for this through the reduction of snout length, which reduces the distance between the input and output force of the jaw lever and maximizes bite force [86]. Furthermore, the observed variations in head morphology can be associated with agonistic interactions. Mice are present in high densities on Cima [32], which could have fostered morphological variation that allows greater bite forces in these populations, a competitive advantage in direct encounters or conflicts [87]. In fact, Cima specimens showed behavioural differences compared to the Fogo populations, and are more aggressive when handled (the authors, personal observations). Alternatively, founder effect and drift may explain these results; however, it would be improbable for two different species to randomly fixate those characters in the same areas.

### 4.3. Evolutionary and conservation perspectives

The morphological variations associated with differential resource use, discussed above, are strong indicators of character displacement. This evolutionary process is defined by an initial interspecific competition for resources, which generates natural selection favouring those individuals best adapted to resource partitioning, which is then reflected in the adaptive divergence of morphological traits [74,88]. The case presented here manifests historical competitive interactions and fulfils several criteria to consider character displacement [88]. *Chioninia delalandii* and *C. vaillantii* are sister species that evolved *in situ* from the same medium-sized size ancestor [31], displaying striking phenotypic variation associated with resource use [27,89]. Apart from the marked difference in body size, the previously reported variation in tooth morphology reflects a clear functional link with different resource exploitation [89]. While *C. delalandii* has sharp unicuspidal teeth, often associated with insectivorous and omnivorous diets, *C. vaillantii* has tricuspid teeth, associated with greater plant consumption [89,90]. In the dietary results presented, the frequencies of plants and invertebrates were similar in the diet of both species, but they may consume plants in different quantities. Additionally, *C. delalandii* allopatric population presented a diet mostly comprised of invertebrates, indicating a resource use shift when in the presence of the larger species. Furthermore, both sympatric *C. delalandii* populations on Fogo and Cima presented similar modifications in head morphology when compared with the *C. delalandii* allopatric population, presenting additional evidence that the sympatric populations of *C. delalandii* have evolved *in situ* head shape characteristics that allow coexistence with the larger competitor *C. vaillantii*. An illustrative example of these dynamics is evident among the diverse *Anolis* lizards from the Antillean Islands, which present remarkable morphological diversity associated with dietary preferences and foraging strategies according to the presence/absence of different competitors [13].

Apart from the above-described dietary and morphological divergence between *C. delalandii* and *C. vaillantii*, additional ecological and evolutionary forces are acting on the Cima populations. This is reflected in their more generalist diet and the significant morphological differences observed between the two species on different islands. This is consistent with an adaptive response to ecological pressures, with both species experiencing similar selective challenges posed by the constrained conditions on Cima. Plastic responses to harsh habitat conditions can be hypothesized to justify these differences [76]. For instance, adaptive changes in morphologies occurred rapidly in the native *Anolis oculatus* (Cope, 1879) when competing with the recent introduction of an alien species in Dominica [91]. However, particularly for *C. delalandii*, genetic differences have been described through the identification of mitochondrial haplotypes unique to these islets that could purely be the result of drift [27].

Finally, all these data provide valuable information for conservation plans. As already reported for birds [32,41], mice are a major threat to reptiles on Cima Islet and eradication plans need to be implemented urgently. It is important to note that *C. vaillantii* is already considered Endangered due to its limited distribution and habitat loss [46] and that *C. delalandii* population on the islet is very reduced (the authors, personal observations) and classified as Data Deficient at the national level [45]. Despite the wide distribution of *C. delalandii*, there is a need to conserve its genetic diversity, as Cima population holds unique genetic and morphological traits that should be protected for the sake of the whole species. In small remote populations, the synergy of genetic and demographic factors significantly increases the probability of extinction [92]. Therefore, given the proven interrelationship between these two species and their fragile/unknown conservation status on the islet, it is important to maintain the balance between them.

## 5. Conclusions

In conclusion, through the use of DNA metabarcoding and morphological analyses, this study highlights the complex interplay between diet, morphology and competitive pressures in shaping the ecological dynamics of sympatric reptile populations, particularly on small islands. The observed variation between sympatric and allopatric populations provides strong evidence for character displacement as an evolutionary strategy allowing resource partitioning and stable coexistence of these species. In addition, increased trophic competition on the small islet further emphasized dietary and morphological variation in sympatric species. The present findings contribute to the understanding of how these species adapt to resource constraints and compete in isolated habitats, providing valuable insights for conservation strategies. For instance, it was revealed the roles that these species play within the particular ecosystem in which they live and the precise trophic items that are essential for their survival. Furthermore, this study highlights the impact of invasive species on the fitness of endemic fauna, emphasizing the need to develop monitoring and control plans, especially in small and fragile islands.

## Data Availability

All data can be found in tables S1 to S4 in the electronic supplementary material [93] and Dryad [94].
